# Oscillatory shear stress promotes angiogenic effects in arteriovenous malformations endothelial cells

**DOI:** 10.1186/s10020-021-00291-6

**Published:** 2021-03-31

**Authors:** Jeong Yeop Ryu, Yun Hyun Kim, Joon Seok Lee, Jeong Woo Lee, Eun Jung Oh, Hyun Mi Kim, Seok-Jong Lee, Jongmin Lee, Sang Yub Lee, Seung Huh, Ji Yoon Kim, Saewon Im, Ho Yun Chung

**Affiliations:** 1 Department of Plastic and Reconstructive Surgery, School of Medicine, Kyungpook National University, 130 Dongdeok-ro, Jung-gu, Daegu, 41944 Korea; 2 Department of Dermatology, School of Medicine, Kyungpook National University, Daegu, 41944 Korea; 3 Department of Radiology, School of Medicine, Kyungpook National University, Daegu, 41944 Korea; 4 Department of Surgery, School of Medicine, Kyungpook National University, Daegu, 41944 Korea; 5 Department of Pediatrics, School of Medicine, Kyungpook National University, Daegu, 41944 Korea; 6 Cell & Matrix Research Institute, School of Medicine, Kyungpook National University, 130 Dongdeok-ro, Jung-gu, Daegu, 41944 Korea; 7 School of Business Administration, Kyungpook National University, Daegu, 41944 Korea

**Keywords:** Shear strength, Arteriovenous malformations, Endothelial cells, Angiopoietin-2, Aquaporin 1, Receptor, Transforming growth factor-beta type I

## Abstract

**Background:**

Vascular endothelial cells (ECs) are subject to continuous shear stress due to blood circulation. Mechanical stress due to high shear flow can also cause arteriovenous malformation (AVM) when ECs respond hyper-sensitively to shear flow. This study was conducted to test the hypothesis that angiogenesis could be promoted in response to mechanical stress via regulation of pro-angiogenic factors in AVM cells.

**Methods:**

ECs were extracted from the tissue samples from six AVM patients and six normal patients. Shear stress at 7 dynes/cm2 were applied for 24 h. Before and after application of shear stress to each group, RT-PCR was performed to access the expression levels of angiopoietin2(AGP2), aquaporin1(AQP1) and TGFβR1. Immunofluorescences was also performed to evaluate the level of protein expressions.

**Results:**

In both normal and AVM tissues, AGP2 and TGFβR1 under the shear stress showed increased expression in the ECs compared to the non-sheared samples. When AVMs and normal arterial vasculature were compared, the expression levels of both AGP2 and TGFβR1 in AVMs were higher when compared to normal arterial vasculature with or without shear stress. Immunofluorescence-based protein analysis also confirmed shear-induced AGP2 and TGFβR1 in both samples of normal and AVM patients.

**Conclusions:**

AVMs exhibited higher sensitivity to shear stress by producing higher expressions of some marked genes and proteins that regulate the endothelial functions upon exposure to shear stress. While the physiological mechanism for AVMs remain elusive, our study shows the plausibility of physical stress imposed by the shearing flow can cause the occurrence of AVMs.

## Background

Vascular anomalies caused by defects in vascular development can lead to abnormalities in various soft tissues, skin, and mucosa. The current classifications of vascular anomalies, proposed by Mulliken and Glowacki, outlined them based on biological processes, clinical features, and histological characteristics (Mulliken and Glowacki 1982), which established the current classification system in the International Society of the Study of Vascular Anomalies (ISSVA). Vascular anomalies can be largely divided into vascular tumors and vascular malformations according to this classification. Vascular malformations are classified as venous, capillary, lymphatic and arteriovenous malformations (AVMs) (Greene et al. 2017).

AVMs occur because of the absence of capillaries between arteries and veins, causing the circulating blood to flow directly from arteries to veins. Blood can either circulate through fistulas which directly connect arteries and veins, or through abnormally connected channel blood vessels from arteries called nidus to the veins. For this reason, AVMs are the most invasive and destructive of the vascular malformations (Kim et al. 2017). Histologically, AVMs are characterized by abnormal channels between arteries and veins, lack of capillaries, and excessive fibrosis (Liu et al. 2010).

Arteriovenous shunting, called nidus, leads to the oxygen deficiency in capillaries, causing tissue ischemia. For AVMs patients, pain, ulcers, bleeding, and tissue destruction appear for this reason. If lesions are present in the mucosal or superficial layer, arterial bleeding may occur when an erosion are developed. In severe cases, AVMs can also progress to the cardiac enlargement and high-output cardiac failure. AVMs can be classified by the Schobinger staging system (Table 1) (Greene et al. 2017).

**Table 1:** Schobinger staging of AVMs

Stage	Clinical findings
I (Quiescence)	Warm, pink-blue, shunting on Doppler
II (Expansion)	Enlargement, pulsation, thrill, bruit, tortuous veins
III (Destruction)	Dystrophic skin changes, ulceration, bleeding, pain
IV (Decompensation)	Cardiac failure

*AVMs* Arteriovenous malformations

Researchers have made many efforts to find the causes of AVMs, but the mechanisms of how and when AVMs develop is not well understood. Five likely causes have been suggested; endothelial cell remodeling, biomechanical activation, hormonal stimulation, extracellular matrix dysregulation, and pericyte dysfunction (Qiao et al. 2019). Among these, during biomechanical activation blood vessels undergo mechanical stimulation caused by muscle movement and/or joint rotation and are constantly sheared by blood flow because of cardiac contractions.

In 1995, there was a report on the association between shear stress and vascular remodeling in brain AVMs (Rossitti and Svendsen 1995). A recent report also revealed the association between brain AVMs and laminar shear stress through systematic review (Lo Presti et al. 2019). In extracranial AVMs, shear stress was measured through Doppler ultrasound and then used for prognosis of AVMs (El Sanharawi et al. 2020). AVMs and shear stress seem to be related in terms of biomechanical activation.

Therefore, we hypothesized that AVMs may develop more frequently if they react more sensitively to mechanical stress compared to normal blood vessels. This study was conducted to test this with the hypothesis that acute, abnormal angiogenesis can occur by reacting sensitively to shear stress.

## Methods

### Isolations and cultures of vascular endothelial cells

This study was approved by the institutional review board (IRB) of Kyungpook National University hospital (IRB number: KNUH 2018-04-020-007) and performed in accordance with the principles of the Declaration of Helsinki. Written informed consent was obtained from all participants. Patient consent was obtained prior to participating in the study. A total of 12 patients (6 patients with AVMs and 6 patients without AVMs) were enrolled in the study. AVM tissues were obtained from 6 patients with AVMs who underwent surgical resection of AVM lesion. Normal arterial vasculature samples were obtained from 6 patients who do not have disease and underwent other surgical procedures (Table 2). These samples were achieved by discarded normal subcutaneous tissues including normal arterial vasculature during surgery. After that, vascular ECs were extracted from the tissue samples. To extract vascular ECs, vascular endothelial tissue was sterilized, cut and placed into Dispase II (GibcoTM Dispase 17105-041, Thermo Fisher Scientific Korea, Seoul, Korea). After incubations for 24 h at 4 °C, the epidermis and dermis were separated. The dermal layers were collected and submerged in 10 ml of HepesBss (GibcoTM HBSS 14170-112, Thermo Fisher Scientific Korea, Seoul, Korea), the supernatant was removed, and 5 ml of Collagenase type I was added. Samples were incubated in a water bath (30 min, 37 °C), after which 10 ml of endothelial cell growth basal medium-2 (EBMTM-2 media CC-3156, Lonza, Walkersville, US) was added and the sample was filtered through a 70 µm nylon filter. The samples were centrifuged at 2000 rpm, for ~ 3 to 5 min, the collected cells were grown in EBMTM-2 media. ECs were grown, sub-cultured and then isolated using the CD31 Microbead kit (Mini&MidiMACS starting kit 130-042-501, Miltenyi Biotec, Auburn, US). This process was repeated for each patient. Molecular biological analysis was performed to confirm that the isolated vascular ECs were pure. First, through reverse transcriptase polymerase chain reaction (RT-PCR), CD31 of marker of vascular endothelial cells was used to compare and analyze. CD31 was strongly expressed in the isolated vascular endothelial cells. In addition, as a result of analysis using CD31 marker (Anti-CD31 antibody ab28364, Abcam, Cambridge, US) through immunofluorescence staining, it was confirmed that most of the cells were strongly expressed in CD31, and also in the morphological part observed in the cell photograph.

**Table 2:** Patient demographics

Patient number		1	2	3	4	5	6
AVMs	Age	13	10	24	41	38	36
	Sex	Male	Male	Male	Female	Female	Female
	Location	Head and Neck	Head and neck	Extremity	Head and neck	Head and neck	Extremity
NA	Age	16	15	28	31	46	42
	Sex	Male	Male	Male	Female	Female	Female
	Location	Head and neck	Head and neck	Extremity	Head and neck	Head and neck	Extremity

*AVMs* Arteriovenous malformations, *NA* normal arterial vasculatures

### Setting the target genes

To identify the difference in the gene expression between the neutral and shear stress environments, and the gene expression of AVMs and normal arterial vasculature, related genes, Angiopoietin2, Aquaporin1, and TGFβR1 were selected and set as target genes for evaluation.

### Flow culture model and quantitative measurement of proper oscillating shear stress

**Fig. 1:**
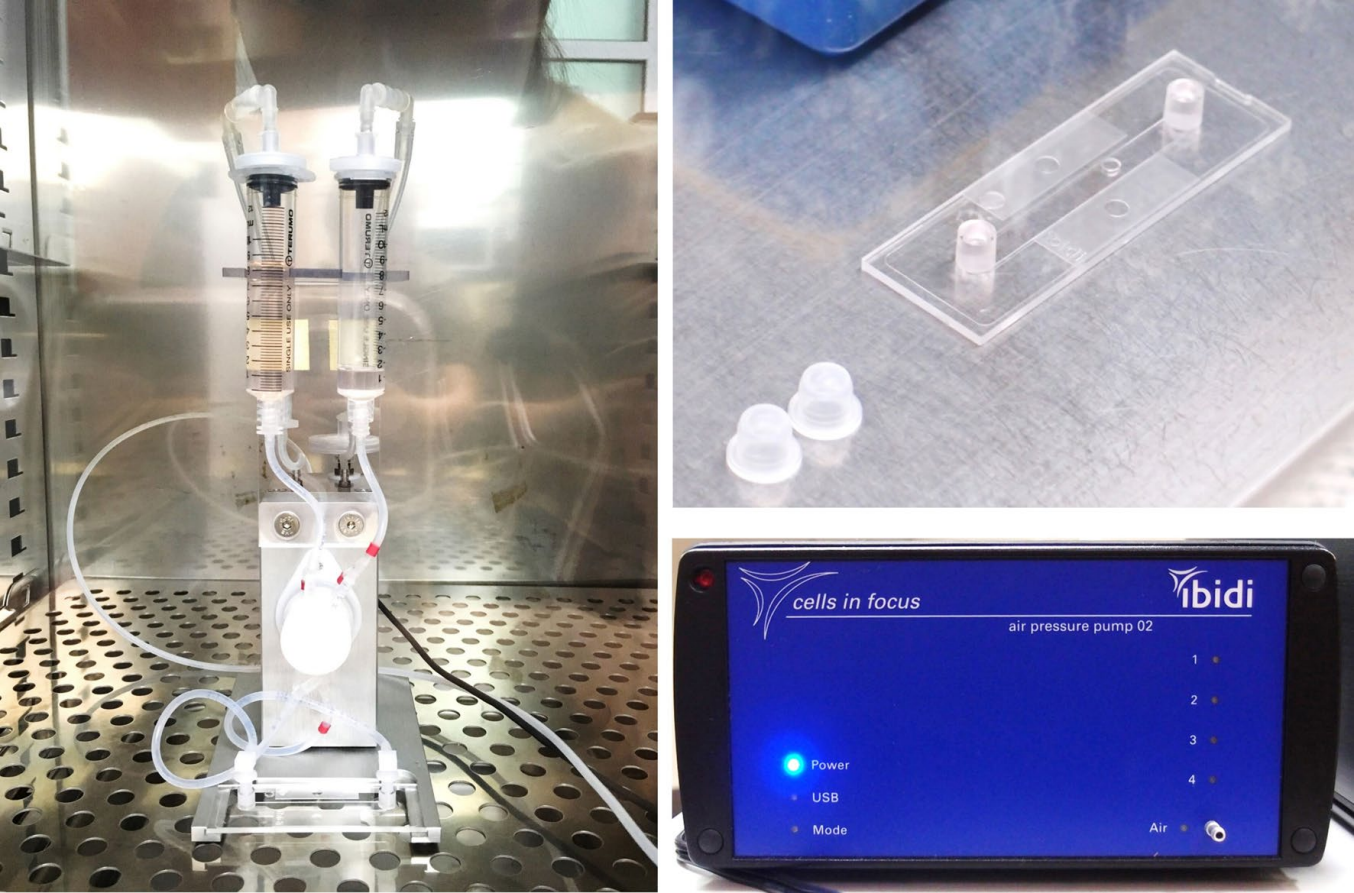
IBIDI flow system for Oscillatory Shear Stress

Flow conditioning of ECs with computerized pump system (ibidi GmbH, Martinsried/Munich, Germany) in Collagen IV-coated μ-Slides I Luer (Fig. 1). The shear stress acted by letting the saline solution flow in the slide within the syringe, then solution was brought back to the opposite syringe. The next flow was shear stressed to the opposite direction. These processes were oscillating shear stress mode among 3 modes (laminar flow mode, pulsatile flow mode, oscillating flow mode) of IBIDI Pump System.

The degree of change in the shape of cells due to shear stress is defined as *M*, the magnitude of the shear stress is *f*, the time at which the stress is applied is *t*, and *k* is a constant, *M* = *kft*. To apply shear stress to the ECs in the samples, the appropriate amount of shear stress (*f*) and time (*t*) when constant shear stress was applied, were quantitatively measured in both groups.

First, in order to find the proper *f*, *t* must be a constant value; therefore, the time to which stress is applied was set to 24 h. The oscillating shear stress manual of the Ibidi flow system was used to measure the minimum shear stress (*f*_*min*_), which shows the beginning of change in cell shape, and the maximum shear stress (*f*_*max*_) just before desorption of cells occurs. Next, in order to measure the maximum time (*t*_*max*_) at which a shear stress can be applied, the *f* value was constant at 4.5 dynes/cm^2^, and the cells were observed under a microscope hourly (0, 6, 12, 24, 48, 72 h) to measure the maximum time needed to cause morphological changes.

#### Cell polarity analysis

Endothelium polarity was quantified using ImageJ software. The polarized angle of each cell was defined as an angle between flow direction and the major axis of the cell body, and angle distribution was plotted to demonstrate the statistical significance. The distribution graph was fitted using a Gaussian function below,

$$f\left(x\right)=\frac{A}{\sigma \sqrt{2\pi }}{e}^{{-\frac{1}{2}\left(\frac{x-\mu }{\sigma }\right)}^{2}}$$

(μ: expected value, σ: standard deviation, A: scale factor).

**Fig. 2:**
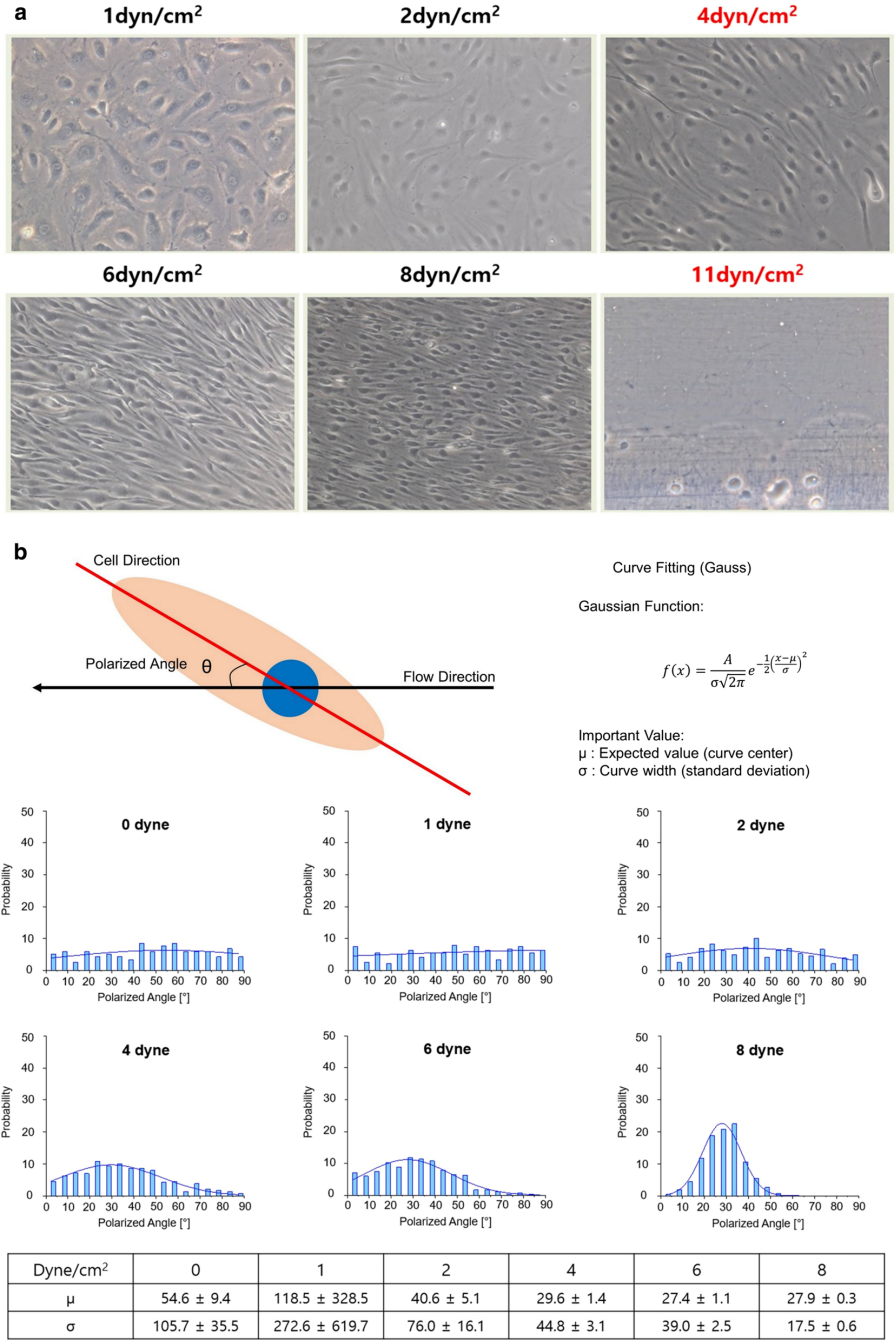
Shear stress dependent microscopic view of endothelial cells (× 20) and quantitative cell polarity analysis. **a** Changes in morphology began at 4 dynes/cm^2^ and cell resorption occurred at 11 dynes/cm^2^. **b** From 4 dynes/cm^2^, cells began to show a Gaussian distribution based on the mean value (μ)

In the angle distribution graph, each data point was normalized by the total cell number and represented as the probability value to ignore the size effect of each sample (Fig. 2).

### RT-PCR (reverse transcription-polymerase chain reaction) & real-time PCR (quantitative PCR)

mRNA expression patterns for the selected genes were analyzed using RT-PCR to validate the expression patterns in vascular ECs with and without shear stress in AVMs and normal arterial vasculature. Tissue RNA was isolated using 900 μl of Trizol, and 1 μg of RNA was collected. RNA was reverse transcribed into cDNA using reverse transcriptase. To investigate the expression level of vascular endothelial cells the following genes were by RT-PCR: Angiopoietin2, Aquaporin1, and TGFβR1. GAPDH was used as the stably expressed control in all samples. Table 3 shows the forward and reverse primers used for gene amplification. In addition, real-time PCR (ABI 7500 Applied Biosystems, Foster City, CA, USA), which can be analyzed quickly, was performed to monitor the products of PCR amplification to accurately quantify the exponential amplification ability, reduce the risk of contamination and eliminated the need for electrophoresis. Mixtures of Sybr Green Master mix (7 µl, ABI 4367659, Applied Biosystems, Foster City, CA, USA), forward primer (1 µl, 10 µM), reverse primer (1 µl, 10 µM), and cDNA (1 µl, 300 ng/µl) were added to the PCR mixture. By repeating the above method, Ct values were measured using real-time PCR and gene expression was analyzed using a comparative Ct method. No-template control (NTC) was included to ensure that the PCR master mix was not contaminated.

**Table 3:** The Primer Sequences for RT-PCR

Gene		Primer sequence (5′–3′)	No. of cycles	Tm (℃)
AGP2	Forward	TGGCTAGTGACCCCCTACAG	32	60
	Reverse	GCTGTGTTCTCTCCAGGCAT		
AGP1	Forward	CTCAGAGGGAATTGAGCACCC	32	57
	Reverse	ATTTGAAGCCCAGGGCAGAA		
TGFβR1	Forward	GCTTAGGGGTGTGGGTCTTC	32	58
	Reverse	AAGCCAAGTTTTCACCCCCA		
GAPDH	Forward	GGAAGGTGAAGGTCGGAGTCA	32	59
	Reverse	GTCATTGATGGCAACAATATCCACT		

*RT-PCR* reverse transcriptase-polymerase chain reaction, *AGP2* Angiopoietin2, *AQP1* Aquaporin1, *TGFβR1* tissue growth factor beta receptor1, *GAPDH* glyceraldehyde-3-phosphate dehydrogenase, *Tm* temperature

#### Relative comparison of gene expression amount before and after applying shear stress

In each sample, the value obtained by subtracting the amount of GAPDH expression from the Ct value of the target gene was defined as dCt, and the dCt._neutral_ value before applying shear stress and dCt._shear_ value after applying shear stress were calculated. For relative comparison of gene expression amounts before and after applying shear stress, the value obtained by subtracting dCt._neutral_ from dCt._shear_ in each sample was defined as ddCt, and the relative gene expression amount, and relative quantity (RQ) from the real-time PCR was calculated by: $RQ={2}^{-ddCt}$ (Bookout et al. 2006; Schmittgen and Livak 2008). This value makes it easier to compare the amount of gene expression in each sample before and after shear stress application. For the relative comparison of gene expression levels before and after applying shear stress for each sample, RQ_avm_ values were calculated in AVMs, and RQ_normal_ values were calculated in normal arterial vasculature. The mean and standard deviation of the RQ_avm_ value of each and RQ_normal_ were determined and compared.

#### Relative comparison of endothelial cells derived from AVMs and normal arterial vasculature

Target gene expression levels in AVMs and normal artery were compared while the conditions for shear stress were matched. The value obtained by subtracting the GAPDH expression amount from the Ct value of the target gene was defined as dCt. After calculating with quantities of gene expression in the AVMs group as: $avm.f\left(dCt\right)={2}^{-dCt.avm}$, and those in normal arterial group as: $normal.f\left(dCt\right)={2}^{-dCt.normal}$, the mean and standard deviation of the relative quantities of each group were calculated. For relative comparison of gene expression amounts between AVMs and normal arterial vasculature, relative quantity (RQ) value was defined and compared as: $RQ=\frac{avm.f(dCt)}{normal.f(dCt)}$. In this method, values were obtained for both states with and without shear stress application.

### Immunofluorescence staining evaluation

Immunostaining of the vascular ECs was performed for the expressed target genes for molecular biological comparison, and to investigate vascular EC function before and after applying shear stress in AVMs and normal arterial vasculature. For staining, an 8-well chamber slide with 70% cell growth was fixed in 4% formaldehyde for 12–24 h, immersed in 0.3% Triton-X100/TBS for 10 min, and washed three times with tertiary distilled water for 1 min each. Blocking buffer immunotech-normal serum was added (10 drops on each cell) and reacted for 5 min. Care was taken not to dry the slide. After removing the serum, the primary antibodies Aquaporin1 (ab168387, Abcam, Cambridge, US), Angiopoietin2 (ab153934, Abcam, Cambridge, US), and TGFβR1 (ab31013, Abcam, Cambridge, US) were diluted (1:100) in antibody dilution solution and added (150 µl each) to the slide, and incubated at 4 °C for 12–24 h. After which, slides were 3 times to react the secondary antibody goat anti-rabbit IgG H & L (Alexa Fluor® 488 ab150077, Abcam, Cambridge, US) for 1 h. After washing three times for 10 min with Tween 20, the vectashield mounting media was added followed by a cover-slide. Cells were observed under a highly sensitive, super resolution, confocal laser scanning microscope: LSM 800 with AiryScan (Carl Zeiss, Oberkochen, Germany).

### Statistical analysis

Statistical analysis was performed using STATA / SE 16.1 (StataCorp, College Station, Texas, USA) based on the data obtained in the description found in step 4. Paired t-tests were performed based on the results before and after applying the shear stress in endothelial cells derived from both AVMs and normal arterial vasculature. Student's *t*-test was performed to compare the results of gene expression in the AVMs group compared to the normal arterial group. Normalization for Student’s t-test was confirmed by Shapiro–Wilk test.

## Results

### Amount and time of proper shear stress

After seeding (6000 cells/cm^2^) of the fibronectin (*1 *µg/ml) coated dish, cells were grown for 24 h. Shear stress was applied for 24 h, the minimum shear stress (*f*_*max*_) per unit area where morphological changes of cells began was 4 dynes/cm^2^, and the maximum shear stress (*f*_*max*_) at which cell detachment occurred was 11 dynes/cm^2^. In the cell polarity analysis, cells began to show meaningful alignment from 4 dynes/cm^2^, assessed based on the mean μ in the Gaussian distribution (Fig. 2).

**Fig. 3:**
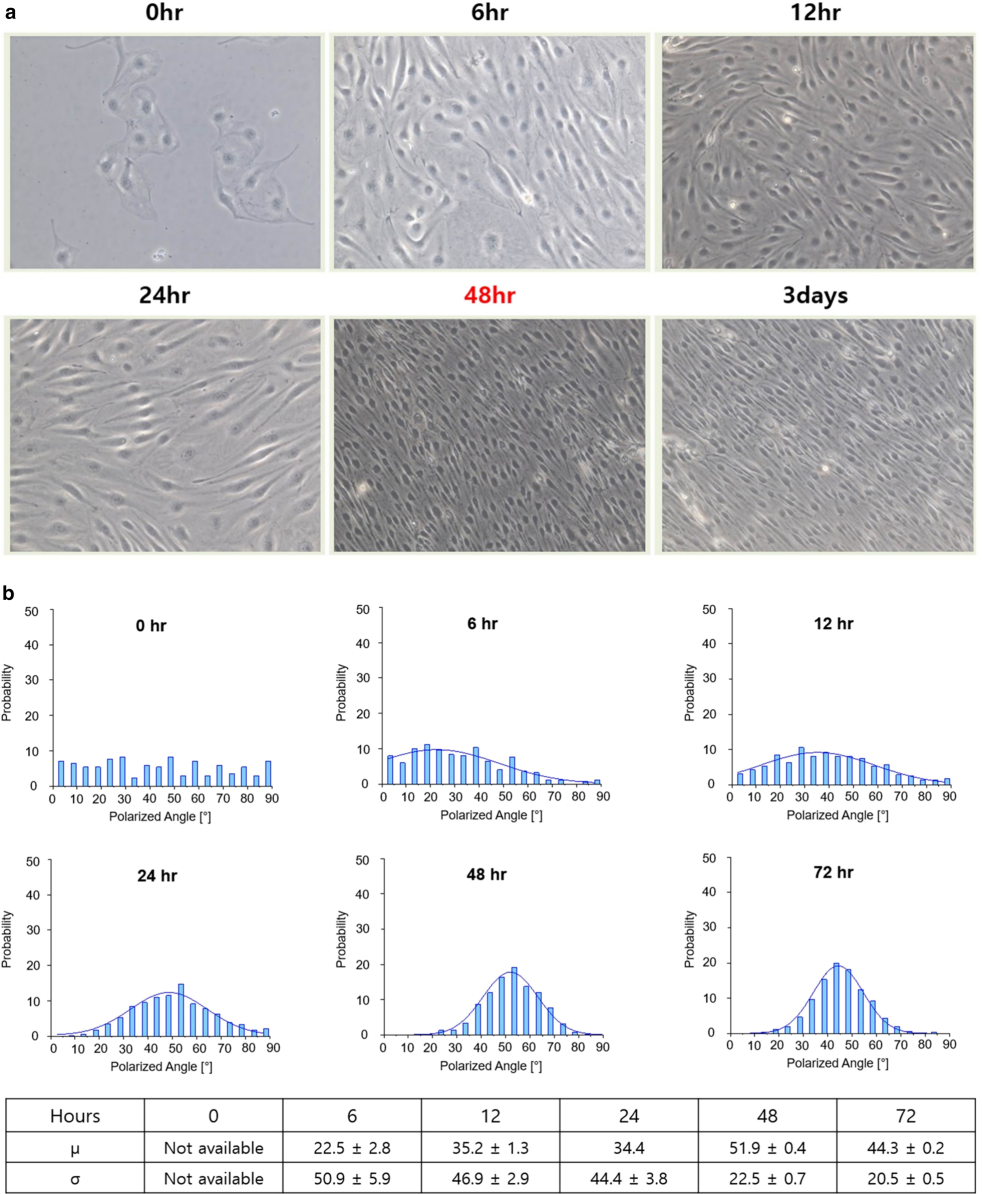
Time dependent microscopic view of endothelial cells (× 20) and quantitative cell polarity analysis. **a** The cells were affected by the shear stress, leading to a change in morphology and which did not differ after 48 h. **b** Cells showed Gaussian distribution based on the mean value (μ) from 6 h. After 48 h, there was no significant difference in the mean value and the standard deviation (σ)

Because the minimum shear stress per unit area at which morphological changes in cells begins was 4 dynes/cm^2^, the shear stress was constant at 4.5 dynes/cm^2^ which is close to the minimum shear stress value in order to obtain the maximum time (*t*_*max*_) to which the shear stress was applied. Vascular ECs were differentiated by morphological changes under the influence of shear stress; importantly, no significant changes in morphology were found after 48 h. In the cell polarity analysis, cells showed noticeable alignment upon exposure to shear stress from 6 h. After 48 h, there was no significant difference in the mean value and the standard deviation (σ). (Fig. 3). Therefore, the maximum time (*t*_*max*_) for applying shear stress was 48 h. Based on this, when performing an experiment, shear stress was applied for 24 h at a size of 7 dynes/cm^2^, which was the median value of minimum shear stress (*f*_*min*_) and maximum shear stress (*f*_*max*_).

### RT-PCR & real-time PCR (quantitative PCR): Angiopoietin2, Aquaporin1, TGFβR1

None of the NTC gave a detectable signal (data not shown).

#### Relative comparison of gene expression amounts before and after applying shear stress

**Fig. 4:**
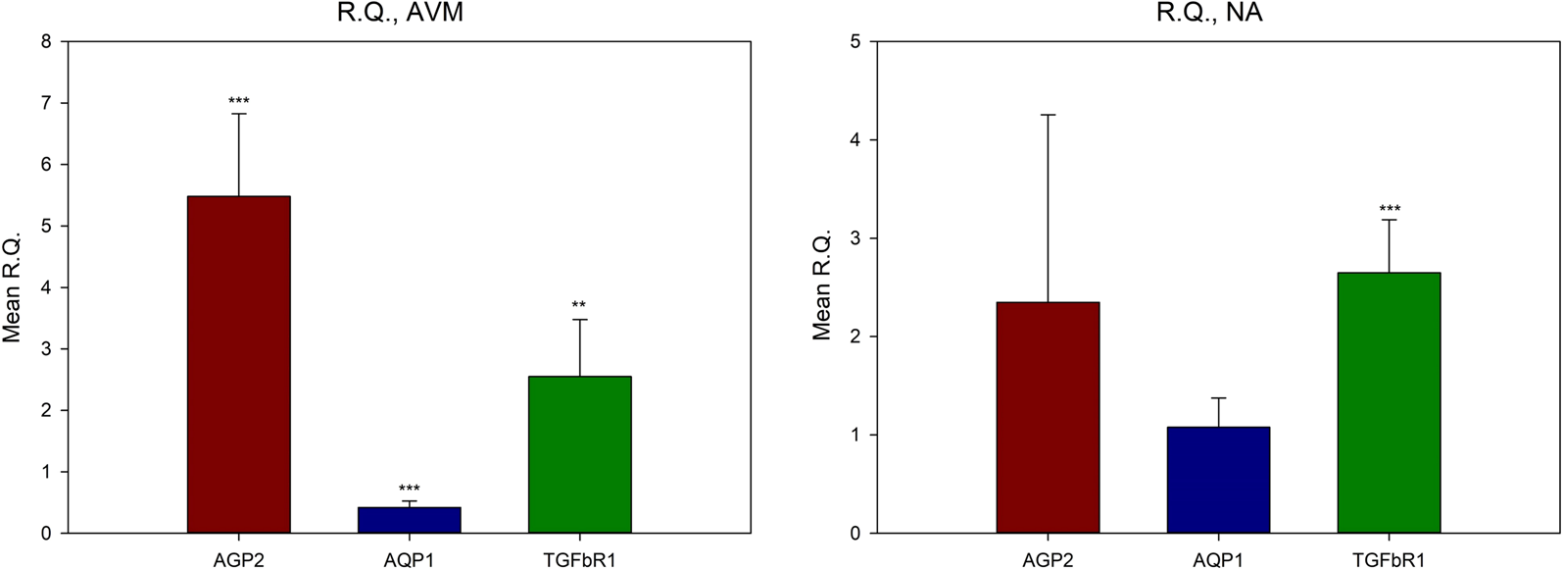
Mean relative quantities (RQs) of AVMs and normal arterial vasculature; comparing shear stress to neutral environments. Compared with neutral environments, both AVMs and normal arterial vasculature showed more gene expressions, except AQP1 when shear stress were applied. *P < 0.05, **P < 0.01, ***P < 0.001. *AVM* Arteriovenous malformations; *R.Q.* relative quantity; *AGP2* Angiopoietin2; *AQP1* Aquaporin1; *TGFβR1* tissue growth factor beta receptor1

The RQ values of Angiopoietin2, Aquaporin1, and TGFβR1 in normal arterial vasculature were 2.350 ± 1.906, 1.078 ± 0.297, and 2.650 ± 0.538, respectively. In AVMs, they were 5.482 ± 1.342, 0.418 ± 0.107, and 2.548 ± 0.929, respectively (Fig. 4). In AVMs RQ value of Aquaporin1was less than 1, therefore the calculation − 1/RQ was used to determine the amount of decrease in gene expression (Schmittgen and Livak 2008). Based on this, the expression level of Aquaporin1, in AVMs was recalculated, and the expression level of the specimen when the shear stress was applied was reduced 2.392 times compared to when shear stress was not applied (Table 4).

**Table 4:** The values of relative quantity for gene expression in shear force and neutral environments, Comparison between shear stress and neutral environments, types of tissues were controlled

Tissue	Gene	Mean RQ	SD	t	P-value
NA	AGP2	2.350	1.906	− 2.2852	0.0711
	AQP1	1.078	0.297	− 0.4473	0.6734
	TGFβR1	2.650	0.538	− 15.6968***	0.0000***
AVMs	AGP2	5.482	1.342	− 9.4514***	0.0002***
	AQP1	0.418	0.107	10.0121***	0.0002***
	TGFβR1	2.548	0.929	− 4.3386**	0.0074**

*AGP2* Angiopoietin2, *AQP1* Aquaporin1, *TGFβR1* tissue growth factor beta receptor1, *AVMs* Arteriovenous malformations, *NA* normal arterial vasculatures, *RQ* relative quantity

*P < 0.05, **P < 0.01, ***P < 0.001

#### Relative comparison of AVMs and normal arterial vasculature

**Fig. 5:**
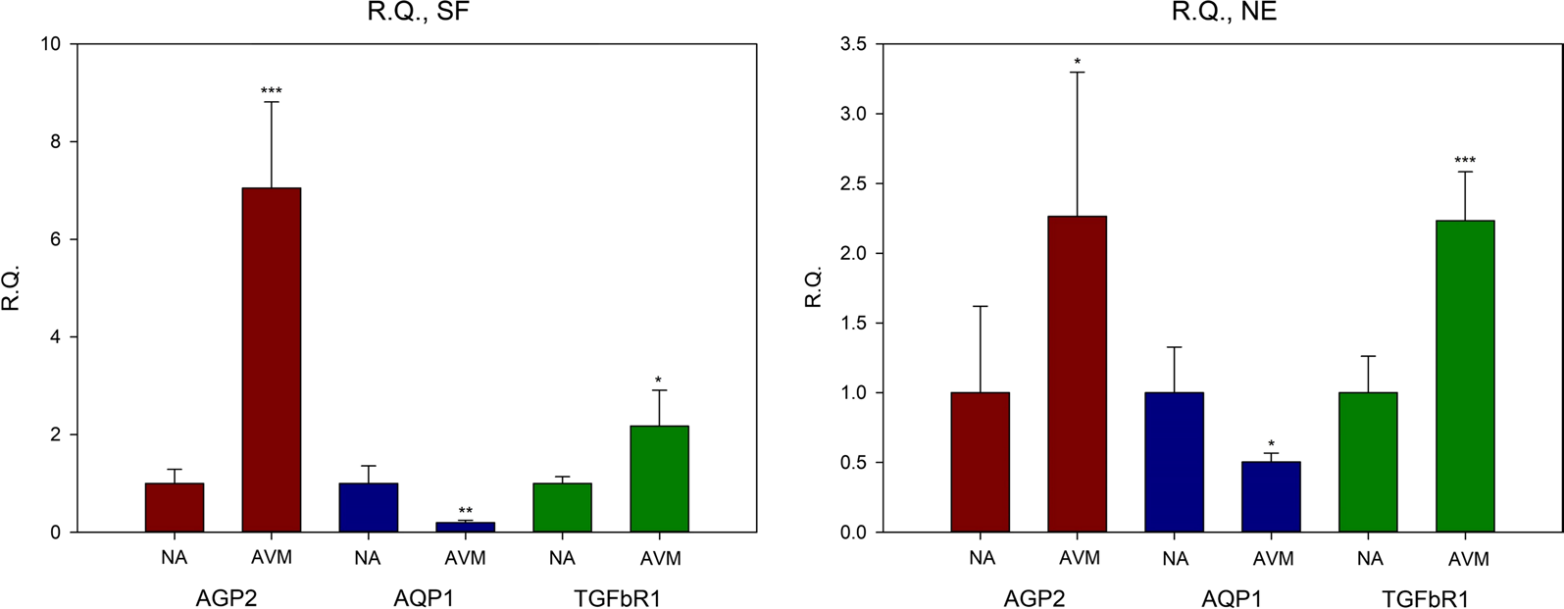
Mean relative quantities (RQs) in shear and neutral environments; comparing AVMs with normal arterial vasculature. Compared with normal arterial vasculature, AVMs showed more gene expression, except AQP1 under both shear and neutral conditions. *P < 0.05, **P < 0.01, ***P < 0.001. *NE* neutral environments; *R.Q.* relative quantity; *NA* normal arterial vasculature; *AVM* Arteriovenous malformations; *AGP2* Angiopoietin2; *AQP1* Aquaporin1; *TGFβR1* tissue growth factor beta receptor1

The RQ values of Angiopoietin2, Aquaporin1, and TGFβR1 in AVMs compared to normal arterial vasculature in the matched state without shear stress were 2.265, 0.505, and 2.234, respectively. The RQ values of Angiopoietin2, Aquaporin1, and TGFβR1 in AVMs compared to normal arterial vasculature when matched with shear stress were 7.052, 0.199, and 2.174, respectively (Fig. 5 and Table 5).

**Table 5:** The values of relative quantity for gene expression in shear force and neutral environments; Comparison between AVMs and normal arterial vasculatures, types of environments were controlled

$f(dCt)$		AVMs (AGP2)	NA (AGP2)	AVMs (AQP1)	NA (AQP1)	AVMs (TGFβR1)	NA (TGFβR1)
NE	Mean	0.251	0.111	3.994	7.912	11.677	5.228
	SD	0.114	0.069	0.499	2.591	1.829	1.371
	RQ	2.265 (0.0278)*		0.505 (0.0132)*		2.234 (0.0000)***	
	t	− 2.5716*		3.6374*		− 6.9119***	
SF	Mean	1.278	0.181	1.658	8.313	29.074	13.373
	SD	0.319	0.052	0.400	2.985	9.836	1.852
	RQ	7.052 (0.0003)***		0.199 (0.0026)**		2.174 (0.0106)*	
	t	− 8.3077***		5.4133**		− 3.8424*	

*AGP2* Angiopoietin2, *AQP1* Aquaporin1, *TGFβR1* tissue growth factor beta receptor1, *AVMs* Arteriovenous malformations, *NA* normal arterial vasculatures, *NE* neutral environments, *SF* Shear force state, *SD* standard deviation, *RQ* relative quantity $RQ=\frac{avm.f(dCt)}{normal.f(dCt)}$

*P < 0.05, **P < 0.01, ***P < 0.001

### Results of immunofluorescence: Angiopoietin 2, Aquaporin 1, TGFb1

To histologically verify the difference in gene expression, immunofluorescence tests for Angiopoietin2, Aquaporin1, and TGFβR1 were performed on vascular ECs before and after applying shear stress.

#### Comparison with and without shear stress

**Fig. 6:**
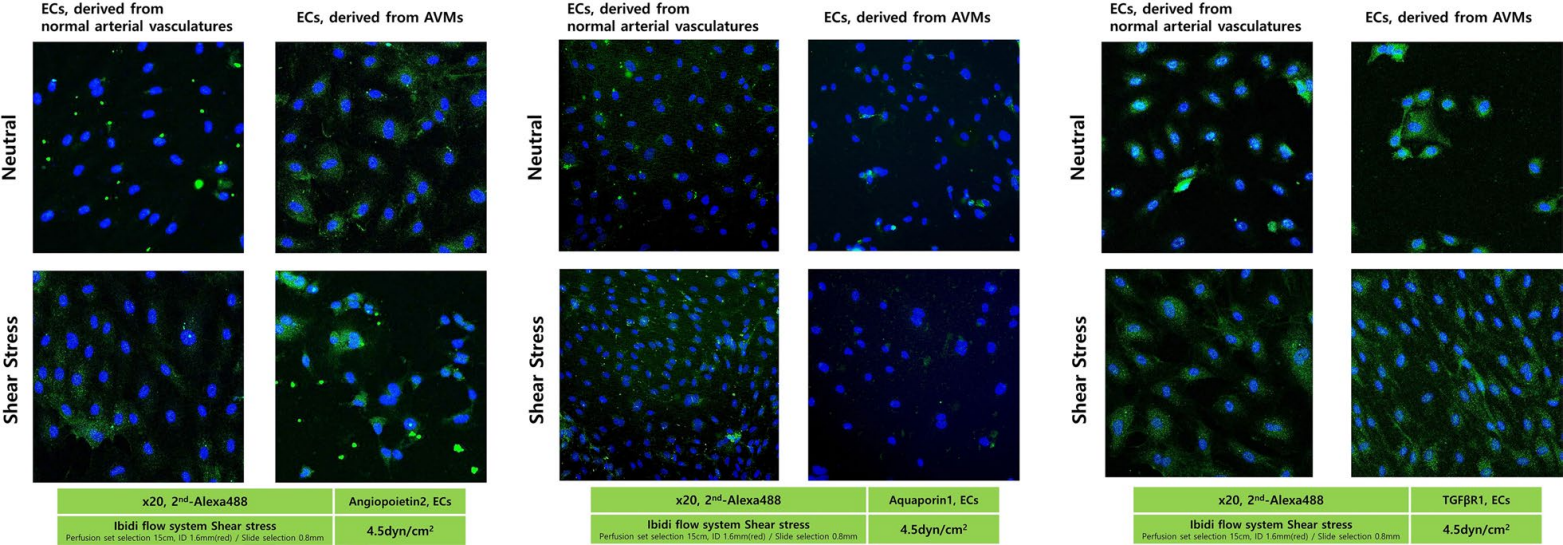
Immunofluorescence for Angiopoietin2, Aquaporin1, and TGFβR1, Endothelial cells (Confocal). In Angiopoietin2 and TGFβR1, shear stress compared with neutral, protein expressions of cytoplasm increased in ECs both normal arterial vasculature and AVMs. Compared with normal arterial vasculature, protein expressions in AVMs increased under neutral and shear stress conditions. Regarding to Aquaporin1, shear stress compared with neutral, protein expressions in normal arterial vasculature increased. There were no significant differences in the degree of cytoplasmic fluorescence staining in endothelial cells from AVMs with and without shear stress application. Compared with normal arterial vasculature, protein expressions in AVMs were less than that observed in normal arterial vasculature under neutral or shear stress conditions. *AVM* Arteriovenous malformations; *ECs* endothelial cells

In normal arterial vasculature the expression of Angiopoietin2, Aquaporin1, and TGFβR1 increased in the degree of fluorescence staining in the cytoplasm with shear stress compared no shear stress. In AVMs the expression of Angiopoietin2 and TGFβR1 increased in the degree of fluorescence staining of the cytoplasm with shear stress compared to no shear stress. Regarding to the expression of Aquaporin1, shear stress compared with neutral, protein expressions in normal arterial vasculature increased. There were no significant differences in the degree of cytoplasmic fluorescence staining in endothelial cells from AVMs with and without shear stress application (Fig. 6).

#### Comparison of normal arterial vasculature and AVMs samples

The degree of fluorescence staining in the cytoplasm for Angiopoietin2 and TGFβR1 increased in the AVMs compared to the normal arterial vasculature samples regardless of the presence or absence of shear stress. When the expression of Aquaporin1 was observed, the degree of fluorescence staining of AVMs was less than that observed in normal arterial vasculature under neutral or shear stress conditions. (Fig. 6).

## Discussion

AVMs were thought to arise due to undesired defects during vascular development (Lee and Chung 2018). Histologically, they were composed of a number of abnormal arteriovenous shunts, and the hemodynamic state in AVMs was varied (Yamada et al. 2004). The increased shear stress on the vascular wall increased the risk of bleeding and induced remodeling of the vessels in AVMs (El Sanharawi et al. 2020). However, the principles and mechanisms were not well understood. In this study, vascular ECs were extracted from human normal vascular tissues and AVMs tissues, and the Angiopoietin2, Aquaporin1, and TGFβR1 gene activation was compared before and after the application of shear stress.

Angiopoietin2 played an important role in angiogenesis through the interaction of angiopoietin (Ang) -tyrosine kinase receptor 2 (Tie2) (Yin et al. 2018). There were reports that Angiopoietin2 contributed to angiogenesis of retinal vessels, and, in animal studies, it was suggested that Angiopoietin2 may promote pathological angiogenesis (Hackett et al. 2002; Lefere et al. 2019).

Aquaporin1 (AQP1) was first purified from a cell membrane called Channel-like Integral membrane Protein of 28 kDa (CHIP28) from red blood cells, and it is now known as an osmolarity-driven water transporter (Smith and Agre 1991). AQP1 was strongly expressed during microvascular proliferation. Endo et al. reported that AQP1 was strongly expressed in the tumor microvasculature in white rats (Endo et al. 1999), and Saadoun et al. confirmed that AQP1 was strongly expressed during microvascular proliferation in human malignant brain tumors. (Saadoun et al. 2002). Vacca et al. reported the relationship of microvascular proliferation and expression of AQP1 in the bone marrow in multiple myeloma (Vacca et al. 2001). Ribatti et al. reported the relationship between microvascular proliferation and expression of AQP1 in embryonic chorioallantoic membranes of chicks (Ribatti et al. 2002). In addition, Kaneko et al. reported a relationship between angiogenesis and AQP1 expression during hypoxia in vitro (Kaneko et al. 2008). These reports provided evidence that AQP1 played a role in microvascular formation and function.

TGF-β was initially reported to have an effect on fibroblasts (Roberts et al. 1983). Since then, the effect has been reported in numerous cell types, such as epidermal, immune, stem, and ECs (Goumans et al. 2009). TGF-β was also found to play an important role in angiogenesis. TGF-β knock-out mice were shown to have angiogenesis and cardiovascular problems (ten Dijke and Arthur 2007). Recent studies have shown that the prevalence of hereditary hemorrhagic telangiectasia (HHT) was higher in people with lower TGF-β levels (Goumans et al. 2009). HHT is an autosomal dominant vascular dysplasia characterized by AVMs in the brain, lungs, liver, and digestive tract. Consequently, errors in the TGF-β signaling system can induce vascular malformations, and may play an important role in the activation and regulation of angiogenesis. Before performing this study, microarray was implemented for investigation of differentially expressed gene as a gene selection method (data was not shown) because AVMs are genetic mutation related disease. Among the various genes with differences, we chose genes that had differences in common with vascular smooth muscle cells (VSMCs), because one of our hypotheses about AVM study is that VSMCs are also work in AVM pathogenesis as well as endothelial cells. So, the above three genes became candidates. And it was considered reasonable to select genes that had differences in common with VSMCs to study the inter-relationship between endothelial cells and VSMCs in the future. In addition, reviews of the literature confirmed that these three genes were related to angiogenesis. Therefore, it was considered reasonable to select genes that had differences in common with vascular smooth muscle cells to study the inter-relationship between endothelial cells and VSMCs in the future. In addition, as mentioned above, reviews of the literature confirmed that these three genes were related to angiogenesis. Therefore, in this study, activation of Angiopoietin2, Aquaporin1, and TGFβR1 were compared to target genes during vascular proliferation.

Shear stress-dependent expression regulation of these genes were also reported. Regarding to the regulation of Foxo-1 and the angiopoietin-2/Tie2 system by shear stress, expressions of AGP2 in epithelial cells were downregulated under the laminar shear stress comparing to static environments (Chlench et al. 2007). Another study also showed the decreased levels of AGP2 expressions under the laminar shear stress (Dixit et al. 2008). Mun et al. showed that laminar shear stress induced the expression of aquaporin1 in endothelial cells involved in wound healing (Mun et al. 2013). Expressions of TGF-β were also induced by laminar shear stress (Walshe et al. 2013; Utsunomiya et al. 2016). However, all these reports were expression regulation by “laminar” shear stress. AVMs have several small diameters of nidi, and because arterial and venous flow are mixed in these nidi, turbulent flows are induced in AVMs. That is why we applied oscillating shear stress in this study. As we know, no report exists about the expression regulation of AGP2, AQP1 and TGFβR1 by oscillating shear stress.

In results of our study, AGP2 expression did not decrease with oscillating shear stress. As mentioned before, in previous reports, it was reported that the expressions of AGP2 were decreased by shear stress. Regarding to this discrepancy, first, tissues which were used in the study were different. Our tissues were not human umbilical vein endothelial cells (HUVECs) but AVMs from real human patients. Second, in the pathological mechanism of AVMs, shear stress cannot be the only reason. The most important pathological mechanism of AVMs is genetic mutations because this disease is congenital vascular malformations. It is not appropriate to simply compare the AGP2 expression pattern in endothelial cells from AVMs with genetic mutations with the HUVEC without any mutations. Third, our study applied the oscillating shear stress. The flows from shear stress have force which consists of magnitude and vector. According to Goettsch et al., long-term low laminar shear stress induced Ang-2 expression and release whereas high laminar shear stress downregulated Ang-2 expression and release (Goettsch et al. 2008). Because oscillating shear stress is a force in the opposite direction of flow regularly, considering a vector, the net of force can be regarded as close to zero or a low shear stress. There is a report that AGP2 was induced at low shear stress, so an appropriate explanation is possible.

AQP1 expression did not increase with additional shear stress, and were not expressed more in AVMs compared to normal arterial vasculature. AQP1 was selected as a target gene in this study because is strongly expressed during microvascular proliferation. Previous reports of AQP1 and microvascular proliferation were mainly focused on angiogenesis in tumors. The angiogenic mechanism of tumors was the ischemic state of tissues due to the rapid growth in tumor size, and this ischemic state induced angiogenesis. AVMs also grow rapidly, but the rate of growth is not as fast as tumors and therefore may not achieve the ischemic condition needed to induce angiogenesis. Therefore, AQP1 may be strongly expressed when angiogenesis occurs during an ischemic condition, but may not be significantly expressed in angiogenesis due to mechanical stimulation, such as shear stress. This may be one of the reasons that the results of AQP1 did not appear as expected. Additional research is needed to investigate this finding.

Shear stress is the force exerted on the inner wall of the tube as the liquid passes through. If the blood vessel is a cylindrical tube and the liquid flowing inside the tube is blood, the shear stress applied to the blood vessel by the blood flow, can be explained by the Hagen-Poiseuille equation. The Hagen-Poiseuille equation is expressed as: $WSS=\frac{32\mu Q}{{\pi D}^{3}}$. WSS = wall shear stress (dynes/cm^2^), µ = blood viscosity (0.035 poise), Q = blood flow rate (ml/s), D = blood vessel diameter (cm). This was the equation used in reports by Zhao et al. and Alaraj et al. (Zhao et al. 2015; Alaraj et al. 2015). According to the equation, the shear stress is proportional to the amount of blood flow per hour, and inversely proportional to the cube of the vessel diameter. Therefore, the greater the amount of blood flow, the greater the shear stress to the blood vessel, and the greater the diameter of blood vessel, the less shear stress is affected. The Vascular ECs form a boundary between the blood flow and the blood vessel wall, and are constantly exposed to shear stress due to blood flow in an in vivo state. Considering why ECs of AVMs were more sensitive to shear stress, vascular ECs develop and differentiate in an environment where shear stress due to turbulent blood flow is always applied. The possibility that these mechanical forces influence morphologically or physiologically aspects of the cells should be considered. In addition, shear stress may induce the synthesis and growth of cells in blood vessels, or may affect the interaction between vascular ECs and other cells (Chiu et al. 2004). Blood vessels are always sheared by the heart's contractions and blood flow. As mentioned before, among 5 causes of AVMs (endothelial cell remodeling, biomechanical activation, hormonal stimulation, extracellular matrix dysregulation, and pericyte dysfunction), shear stress can be considered as biomechanical activation. Therefore, it shear stress may be one of the causes in the development of AVMs. The results of this study support that the shear stress in biomechanical activation may be one of the causes of AVMs.

## Conclusions

In this study, it was shown that the AVMs have a high sensitivity to shear stress. As a result, AVMs had more angiogenic potential than normal arterial vasculature, leading us to believe the physiological correlation between shear stress and the pathogenesis of abnormal angiogenesis in AVMs. This study was an analysis of gene and protein expressions of cells from normal versus AVMs in the presence or absence of shear stress. Our results may shine light in identifying factors leading to AVMs, and may be useful for future genetic diagnosis and treatment.

## Data Availability

The datasets generated and analyzed during the current study are not publicly available due to the study’s data access policies. but are available from the corresponding author on reasonable request.

## Abbreviations

EC: Endothelial cell
VSMCs: Vascular smooth muscle cell
AVM: Arteriovenous malformation
AGP2: Angiopoietin2
AQP1: Aquaporin1
TGFβR1: Transforming growth factor-beta receptor 1
IRB: Institutional review board
RT-PCR: Reverse transcription-polymerase chain reaction
RQ: Relative quantity
HHT: Hereditary hemorrhagic telangiectasia
